# Arbuscular mycorrhizal fungi in the soil using cover crops with and without nitrogen addition

**DOI:** 10.3389/fpls.2022.1054484

**Published:** 2022-11-11

**Authors:** Jadson Belem de Moura, Maria Lucrécia Gerosa Ramos, Maria Luiza de Freitas Konrad, Orivaldo José Saggin Júnior, Walter Quadros Ribeiro Junior, Arminda Moreira de Carvalho, Luana de Miranda Santos, Rodrigo Fernandes de Souza, Luiz César Lopes Filho, Sandro Dutra e Silva

**Affiliations:** ^1^ Graduate Studies in Social, Technological and Environment Science, Evangelical University of Goiás, Anápolis, Brazil; ^2^ Sedmo—Soil Research Group, Ecology and Dynamics of Organic Matter, Evangelical College of Goianésia, Goianésia, Brazil; ^3^ Faculty of Agronomy and Veterinary Medicine, University of Brasilia, Brasilia, Brazil; ^4^ Federal University of Tocantins, Palmas, Tocantins, Brazil; ^5^ Brazilian Agricultural Research Company, National Center for Research in AgroBiology (CNPAB), Rio de Janeiro, Brazil; ^6^ Cerrados Agricultural Research Center (CPAC), Embrapa Cerrados, Brasilia, Brazil; ^7^ Graduate Studies in Natural Resources of the Cerrado, State University of Goiás, Anápolis, Brazil

**Keywords:** savannas ecosystem, no tillage system, planting systems, cover plants, Cerrado (Brazil)

## Abstract

This study aimed to evaluate the occurrence of mycorrhizal fungi and glomalin content in soil under different cover crops with and without the application of nitrogen in the cover. The following cover plants were used: Crotalaria juncea (*Crotalaria juncea* L.), wild beans from Ceará (*Canavalia brasiliensis* Mart. ex Benth.), Guandú ‘BRS mandarin’ [*Cajanus cajan* (L.) Millsp.], millet ‘BR05’ [*Pennisetum glaucum* (L.) R.Br.] and sorghum ‘BR 304’ [*Sorghum bicolor* (L.) Moench]. The absolute control of the experiment was the treatment without the use of cover crops, that is, the vegetation of spontaneous occurrence in the area. The experimental design was randomized blocks in subplots with three replications. Spore density, mycorrhizal colonization rate, easily extractable glomalin, and species present in the rhizosphere of the cover crops were determined. No differences were found in the diversity of mycorrhizal fungi associated with the different cover crops studied or in the values of spore density, root colonization, or glomalin content. Nitrogen application did not influence the mycorrhizal activity in the investigated cover crops. The most frequent species associated with cover crops were *Scutellospora pellucida* and *Scutellospora persica* in *C. juncea*; *Gigaspora* sp. on Sorghum; *Glomus macrocarpum* in Guandu; *G. macrocarpum* and *Glomus clavisporum* in millet; and *Glomus microaggregatum* and *Glomus tortuosum* in Spontaneous Vegetation.

## Introduction

Conservation cultivation systems, such as no-tillage, are at the forefront of sustainable soil management and water resources (Borie et al., 2006). One of the premises for the success of this system is soil vegetation cover that brings numerous benefits, such as erosion protection, intake of organic material into the soil, nutrient cycling, and maintenance of an ideal microclimate for microbial development (Borie et al., 2006; Abdollahi et al., 2014; Bongiorno et al., 2019).

Cover plants are responsible for soil protection in these systems and can influence the diversity and richness of the rhizospheric microorganism community in the soil (Alguacil, 2015). Arbuscular mycorrhizal fungi (AMF) are one of the most important groups of edaphic organisms, and their activity is closely linked to plant health and can be used as a bioindicator of environmental quality in different soil management systems (Borie et al., 2006; Alguacil et al., 2008; Alguacil, 2015; Moura et al., 2017; Moura and Cabral, 2019).

Different species of cover crops are used in sustainable agricultural management in the Brazilian Cerrado region, which is considered one of the main agricultural frontiers of today (Hendrix et al., 1988; Beuchle et al., 2015). Among them, some forages of the Fabaceae and Poaceae families stand out for being adapted to the edaphoclimatic conditions of the region, in addition to bringing benefits to agroecosystems such as biological nitrogen fixation and low decomposition velocity (Bowles et al., 2017; Boutasknit et al., 2020).

Nitrogen availability is another factor to be considered when handling vegetation cover in the soils of tropical regions, such as the Cerrado. Nitrogen contributes to plant biomass protein, providing more efficient soil protection, and when managed before planting, vegetation cover promotes more efficient nutrient cycling for subsequent crops (Alves et al., 2006; Ahiabor et al., 2007). This makes the practice of nitrogen fertilization common during the management of cover crops in the Cerrado.

Given its importance in the regional development of agriculture in the Cerrado region, understanding the biodiversity of arbuscular mycorrhizal fungi associated with the main cover crops used in conservation systems is of fundamental importance for the effective management of management practices in this region (Moura and Cabral, 2019; Aker et al., 2022; Durigan et al., 2022).

The objective of this work was to evaluate the occurrence of mycorrhizal fungi and glomalin content in soil under different cover crops cultivated after corn crops, with and without nitrogen (N) addition in the cover.

## Material and methods

The corn experiment (*Zea mays* L.) in succession to the cover crops was planted in 2005 in an area of Embrapa Cerrados in Planaltina, DF (15°35’30”S, 47°42’30”W, at 1,000 m altitude). Between 1995 and 2005, the area was in the field. The soil of the experimental area is classified as typical dystrophic Red Latosol, Moderate A, and tropical Cerrado phase, with smooth wavy relief, and clayey texture (Santos et al., 2013). The evaluations were carried out in the agricultural years 2009/2010 and 2010/2011. The chemical analysis of the soil collected in the 0–10 cm depth layer at the end of the 2010/2011 agricultural year, according to Claessen (1997), presented the following results: pH 5.9 in H2O; 26.3 g kg^−1^ organic matter; 8.3 mg dm^3^ of P-Mehlich1; 0.07 cmolc dm^3^ of Al; 3.6 cmolc dm^3^ ca + mg; and 0.2 cmolc dm^3^ of K. The climate is classified as a tropical station, Aw, according to Köppen, and is characterized by two well-defined seasons (dry and rainy) and the occurrence of periods of drought during the rainy season (Cardoso et al., 2015). In April 2010, the following plant species were sown in the area for soil cover: Crotalaria juncea (*Crotalaria juncea* L.), ceará wild beans (*Canavalia brasiliensis* Mart. ex Benth.), Guandu “BRS mandarin” [*Cajanus cajan* (L.) Millsp.], Millet “BR05” [*Pennisetum glaucum* (L.) R.Br.] and Sorghum “BR 304” [*Sorghum bicolor* (L.) Moench]. The absolute control of the experiment was the treatment without the use of cover crops, that is, the vegetation of spontaneous occurrence in the area (Spontaneous Vegetation). The plant density was 20 plants per linear meter for *C. juncea*, Guandu, sorgo, wheat, and *Brachiaria ruziziensis*; 40 plants per linear meter for millet and turnip; and 10 plants per linear meter for wild beans. The spacing between sowing lines was 0.5 m for all plant species (Carvalho and Amabile, 2006). The sowing of the cover crops was carried out directly on the crop remains of corn cultivated in the previous agricultural year, crop 2009/2010. The experimental design of randomized blocks was used, with subdivided plots and three replications. The plots were represented by the cover species (12 × 8 m), and the subplots (12 × 4 m), by the cutting times in flowering and physiological maturation, which totaled 3,420 m^2^ of total area.

The experiment presented the same sequence of crops (cover crops in succession to corn) until 2012. In the flowering period of the cover plants, roots and rhizosphere soil were collected at a depth of 0–20 cm with Dutch trade in June 2012. From each of the treatments, a composite sample consisting of three subsamples of random points in the subplot was taken, and these composite samples were homogenized and transported to the Soil Biology Laboratory of Agronomy-UnB.

The spores were extracted from the soil using 50 ml of each composite sample by the wet sieving technique (Gerdemann and Nicolson, 1963), followed by centrifugation in water and then in a 50% sucrose solution. Subsequently, quantification and separation of spores were performed by morphological characteristics under a stereoscopic bilocular magnifying glass. To determine the percentage of colonization, the roots were clarified and cordoned with 0.05% Trypan Blue in lactoglycerol (Phillips and Hayman, 1970), and the colonization evaluation was performed under a stereoscopic microscope, following the technique of intersection of the quadrants (Giovannetti and Mosse, 1980). The Bradford method was used to determine the easily extractable reactive protein (or easily extracted glomalin) according to the methodology of Wright and Upadhyaya (Wright and Upadhyaya, 1996). One gram of soil was weighed in Falcon tubes with a capacity of 50 ml. Duplicates were made of each soil sample. Eight milliliters of 20 mM sodium citrate buffer solution, pH 7.0, was added to each tube, which was autoclaved for 30 min at 121°C. Then, the vials were centrifuged at 5,000 rpm for 10 min. To determine the concentration of glomalin, 50 μl of the extract was pipetted into the test tube, and 1 ml of the Bradford reagent was added to the tubes. After this procedure, the tubes were taken for vortex agitation. After 10 min, the absorbance reading was made in a spectrophotometer at 595 nm.

For the identification of AMF species from morphological characteristics, spores were separated according to their morphotypes and mounted on blades with pure polyvinyl-lactoglycol (PVLG) and PVLG mixed with Melzer (1:1 v/v). The identification of mycorrhizal fungi species was performed in the Mycorrhizas Laboratory of Embrapa Agrobiologia, following the descriptions of the reference cultures present in the International Culture Collection of Arbuscular and Vesicular-Arbuscular Mycorrhizal Fungi (INVAM, 2022).

The data were submitted to variance analysis by the Assistat (e Silva, 2008) analyses of canonical correspondence were performed by the Past (Hammer, 2021) software. Spore density variables and the rate of mycorrhizal colonization were determined by a 5% Tukey test. The presence of identified genera was used as the parameter for multivariate analysis. Data were normally distributed. Canonical correspondence analysis was performed based on the presence/absence data of AMF in samples or sites.

## Results

The analysis of variance (**Table 1**) showed that there was a statistically significant difference only for the spore density, which showed a minimally significant difference in relation to cover crops. **Table 2** presents spore density data (in 50 ml of soil) of cover crops under nitrogen application.

**Table 1:** Analysis of variance of easily extracted glomalin contents, mycorrhizal colonization rate and number of soil spores.

Source of Variation	Test F		
	Glomalin	Colonization	Spores
Vegetation Cover (F1)	2.4862 ns	1.6936 ns	4,8520*
Nitrogen (F2)	0.2522 ns	0.0106 ns	0.2844 ns
Interaction (F1 × F2)	1.2453 ns	1.0037 ns	1.8369 ns

*Significant at the 5% probability level (p <.05).

ns, not significant (p ≥.05).

**Table 2:** Spore density (n°/50** ml**) of cover crops after maize cultivation, with and without nitrogen fertilization in top-dress, in maize. Log transformed data (x + 1).

Cover Plant	Nitrogen fertilization		Average
	With N	Without N	
Crotalaria	552.33	833.00	692.67a
Wild beans from Ceará	560.67	465.00	512.83ab
Sorghum	411.33	680.33	545.83ab
Guandu	921.00	587.67	754.83a
Millet	235.50	398.00	316.74b
Spontaneous Vegetation	495.50	443.00	468.25ab
Average	529.05A	567.83A	
CV%- cover crops = 5.43 CV%- Nitrogen fertilization = 5.40			

Means followed by the same uppercase letter in the rows and lowercase in the columns do not differ from each other by the Tukey test (p <0.05)

The soil under Guandú and *C. juncea* had the highest number of spores compared with the other treatments, and the soil under millet had the lowest spore density values. The treatments using wild beans from Ceará, sorghum, and spontaneous vegetation showed intermediate values of spore density (**Table 2**).

There was no significant difference between the cover crops or between the application or not of cover N in the maize crop in mycorrhizal colonization, which varied between 63.07% and 78.97% among the different plant species (**Table 3**), and there was no significant effect of cover crops and N application in topdressing on easily extractable glomalin (**Table 4**).

**Table 3:** Mycorrhizal colonization (%) of cover crops after corn cultivation, with and without Nitrogen fertilization in cover, in corn.

Cover Plant	Nitrogen fertilization		Average
	With N	Without N	
Crotalaria	75.55	77.69	76.62
Wild beans from Ceará	63.62	62.51	63.07
Sorghum	74.62	83.33	78.97
Guandu	75.55	73.62	74.59
Millet	71.74	81.67	76.70
Spontaneous Vegetation	68.48	71.30	69.89
Average	71.60	75.02	
CV%- cover crops = 14.71; CV%- Nitrogen fertilization = 13.99.			

**Table 4:** Easily extractable glomalin (mg kg soil^−1^) from cover crops, after corn cultivation, with and without nitrogen fertilization in cover, in corn.

Cover Plant	Nitrogen fertilization		Average
	With N	Without N	
Crotalaria	1.96	1.42	1.69
Wild beans from Ceará	1.76	1.77	1.74
Sorghum	1.71	2.23	1.99
Guandu	1.71	2.03	1.87
Millet	1.48	1.56	1.51
Spontaneous Vegetation	1.57	1.57	1.57
Average	1.70	1.76	
CV%- cover crops = 16.12; CV%- Nitrogen fertilization = 22.08.			

**Figure 1:**
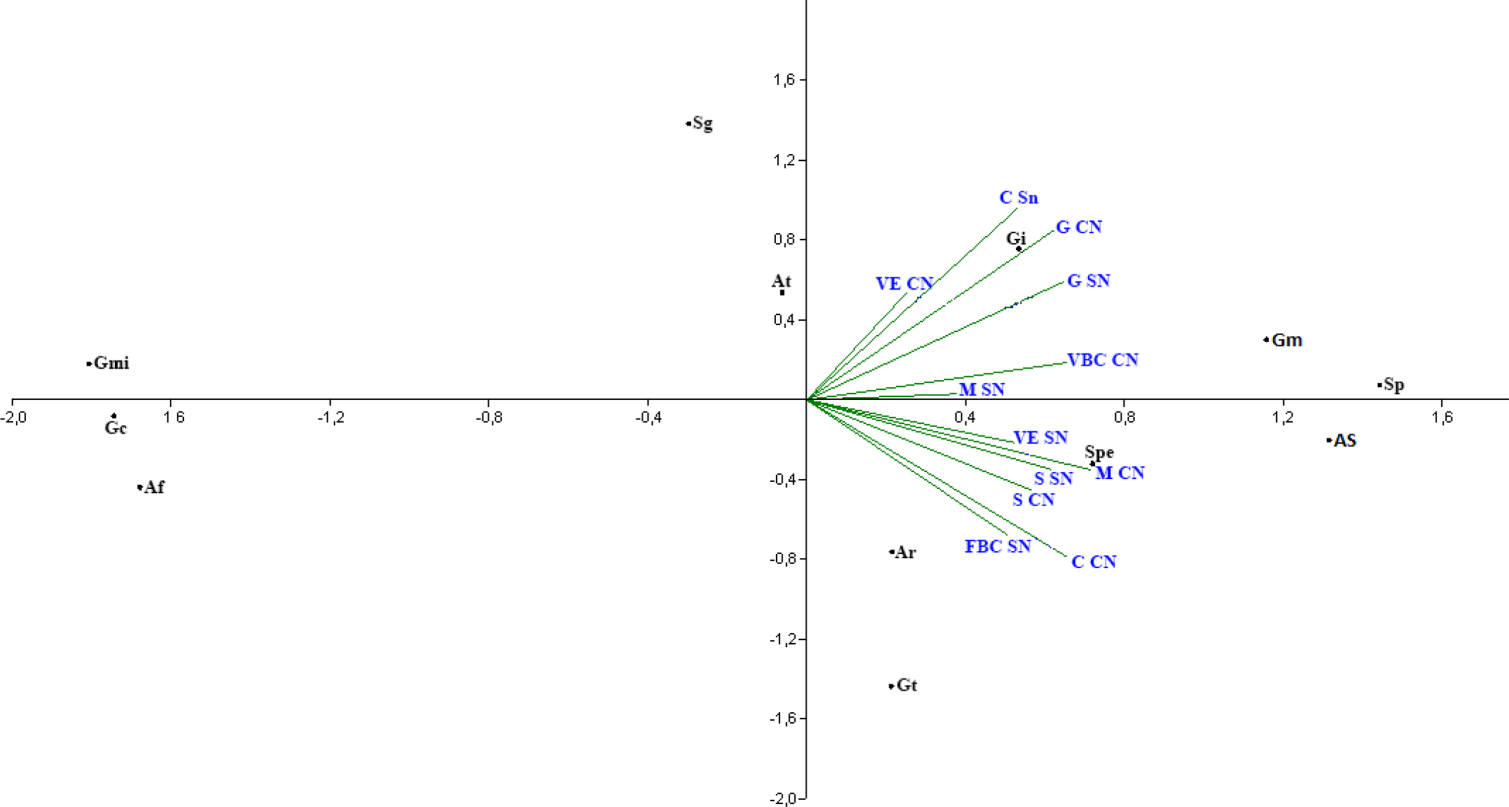
Analysis of principal components of the frequency of arbuscular mycorrhizal fungi species found in the rhizosphere of Crotalaria (C), bean—bravo-do-ceará (FBC), sorghum (S), guandú (G), millet (M) and spontaneous vegetation (VE) with and without nitrogen addition. (Af, *A. foveata*; At, *A. tuberculata*; Ar*, Ar. leptoticha*; Gc, *G. clarum*; Gmi, *G. microaggregatum*; Gt*, G. tortuosum*; Gi*, Gigaspora* sp.; Sg, *S. gregaria*; Sp, *S. pellucida*; Spe, *S. persica*; *Gm*, *G. macrocarpum*; *As*, *A. scrobiculata*).

The principal component analysis points to the correlation of cover plant species with the presence and frequency of associated arbuscular mycorrhizal fungi species in the rhizosphere. All the cover plant species studied show similar behavior in relation to the frequency of arbuscular mycorrhizal fungi species that colonize the rhizosphere of these cover plants (**Figure 1**).

The *C. juncea* treatment and application of nitrogen as a top dressing in the predecessor culture differs from the *C. juncea* treatment without nitrogen application in relation to the most frequent arbuscular mycorrhizal fungi species in rhizospheric soil (**Figure 1**). The species *A. tuberculata*, *G. macrocarpum*, and *Gigaspora* sp. are more commonly present in the rhizosphere of *C. juncea* with nitrogen application, while *Ar. leptoticha*, *G. tortuosum*, and *S. persica* are closer to the treatment without N application in topdressing in corn.

The soil under millet, regardless of nitrogen application, showed an approximation of the species *S. persica*, *Ar. leptoticha*, and *G. tortuosum*. The species *G. microaggregatum*, *S. gregaria*, *G. clavisporum*,and *A. foveata*, according to the analysis of principal components (**Figure 1**), were not frequently found in the rhizosphere of the cover plants analyzed, indicating that these species were present by chance in the rhizospheric soil of cover crops.

**Table 5** presents species of arbuscular mycorrhizal fungi found in the rhizosphere of Crotalaria (C), the wild bean from Ceará (FBC), sorghum (S), guandú (G), millet (M), and spontaneous vegetation (VE) with and without the addition of nitrogen in no-tillage in the cerrado.

**Table 5:** Species of arbuscular mycorrhizal fungi found in the rhizosphere of Crotalaria (C), wild bean from Ceará (FBC), sorghum (S), Guandú (G), millet (M) and spontaneous vegetation (VE) with and without addition of nitrogen in planting straight into the savannah.

Species	C	FBC	S	G	M	VE	C	FBC	S	G	M	VE
	With N						Without N					
*A. foveata*	−	−	−	−	−	−	−	+	−	−	−	−
*A. scrobiculata*	+	+	+	+	+	−	+	+	+	+	+	+
*A. tuberculata*	−	−	+	+	+	−	+	−	+	+	−	−
*Ar. leptoticha*	+	−	+	−	+	+	−	+	−	+	+	+
*G. clavisporum*	−	−	−	−	−	−	−	−	−	−	+	−
*G. tortuosum*	+	+	+	−	+	−	−	+	+	−	−	+
*G. microaggregatum*	−	−	−	−	−	+	−	−	−	−	−	−
*G. macrocarpum*	+	+	−	+	+	+	+	+	+	+	+	+
*Gigaspora sp*	−	+	+	+	+	+	+	+	−	+	+	−
*S. gregária*	−	+	−	+	−	+	+	−	−	+	−	+
*S. pérsica*	+	+	+	+	+	+	−	+	+	+	−	−
*S. pellucida*	+	+	+	+	+	+	+	+	+	+	+	+
Total species	6	7	7	7	8	7	6	8	6	8	6	6

A, Acaulospora; Ar, Archaeospora; G, Glomus; S, Scutellospora.

The soil under the cover crops Guandú and wild beans from Ceará, in addition to spontaneous vegetation with nitrogen application and the same treatment without nitrogen in cover, showed similar behavior in relation to the presence of AMF species. The species *G. macrocarpum*, *Gigaspora* sp., *S. pellucida*, and *A. tuberculata* were more frequent in the rhizospheres of plants under nitrogen application, and *A. scrobiculata*, *S. persica*, *Ar. leptotic*, and *G. tortuosum* are closer to the species without nitrogen application.

The species *Ar*. *leptoticha* and *S. persica* were only identified in the rhizosphere of *C. juncea* under nitrogen application, while *A. tuberculata*, *S. gregaria*, and *Gigaspora* sp. were only associated with *C. juncea* without nitrogen application. The species *A. scrobiculata*, *G. macrocarpum*, and *S. pellucida* were identified in the rhizosphere of *C. juncea* with and without nitrogen application.

*The species S. gregaria* occurred only in *the* rhizosphere of wild beans from Ceará with the application of nitrogen in topdressing in corn. In the treatment without the application of nitrogen, *A. foveata* and *Ar. leptoticha* were identified. The *species A. scrobiculata*, *G. tortuosum*, *G. macrocarpum*, *Gigaspora* sp., *S. persica*, and *S. pellucida* were identified in the rhizosphere of wild bean plants, with and without nitrogen application.

The species *G. macrocarpum* occurred only in the sorghum rhizosphere without nitrogen application in corn, and the species that occurred in the soil with sorghum use only under nitrogen application were the following: *Ar. leptoticha* and *Gigaspora* sp. The species *A. scrobiculata*, *A. tuberculata*, *G. tortuosum*, *S. persica*, and *S. pellucida* were identified in the sorghum rhizosphere, with and without application of nitrogen in topdressing in corn.

Among the species identified in the rhizosphere of Guandú, *A. scrobiculata*, *A. tuberculata*, *G. macrocarpum*, *Gigaspora* sp., *S. gregaria*, *S. persica*, and *S. pellucida occurred* both in plots with nitrogen addition and without N application in corn cover. The species *Ar. leptoticha* was found only in areas of Guandú without nitrogen application.

In millet rhizospheric soil, with and without nitrogen application in topdressing in the corn predecessor crop, the following species were identified: *A. scrobiculata*, *Ar. leptoticha*, *G. macrocarpum*, *Gigaspora* sp., and *S. pellucida*. In treatments without nitrogen application in topdressing in corn, the species *G. clavisporum was still identified*, while in areas with nitrogen application in topdressing, the presence of the species *A. tuberculata*, *G. tortuosum*, and *S. persica* were detected. In the rhizosphere of the plants of spontaneous occurrence in the area (spontaneous vegetation), with the application of nitrogen in the corn cover, the species *G. microaggregatum*, *Gigaspora* sp., and *S. persica*, which occurred only in this treatment. On the other hand, in the treatments without the application of nitrogen in topdressing in the corn crop, the identified species were *A. scrobiculata* and *G. tortuosum*. The species *Ar. leptoticha*, *G. macrocarpum*, *S. gregaria*, and *S. pellucida* were identified in both treatments, with and without nitrogen application.

**Figure 2:**
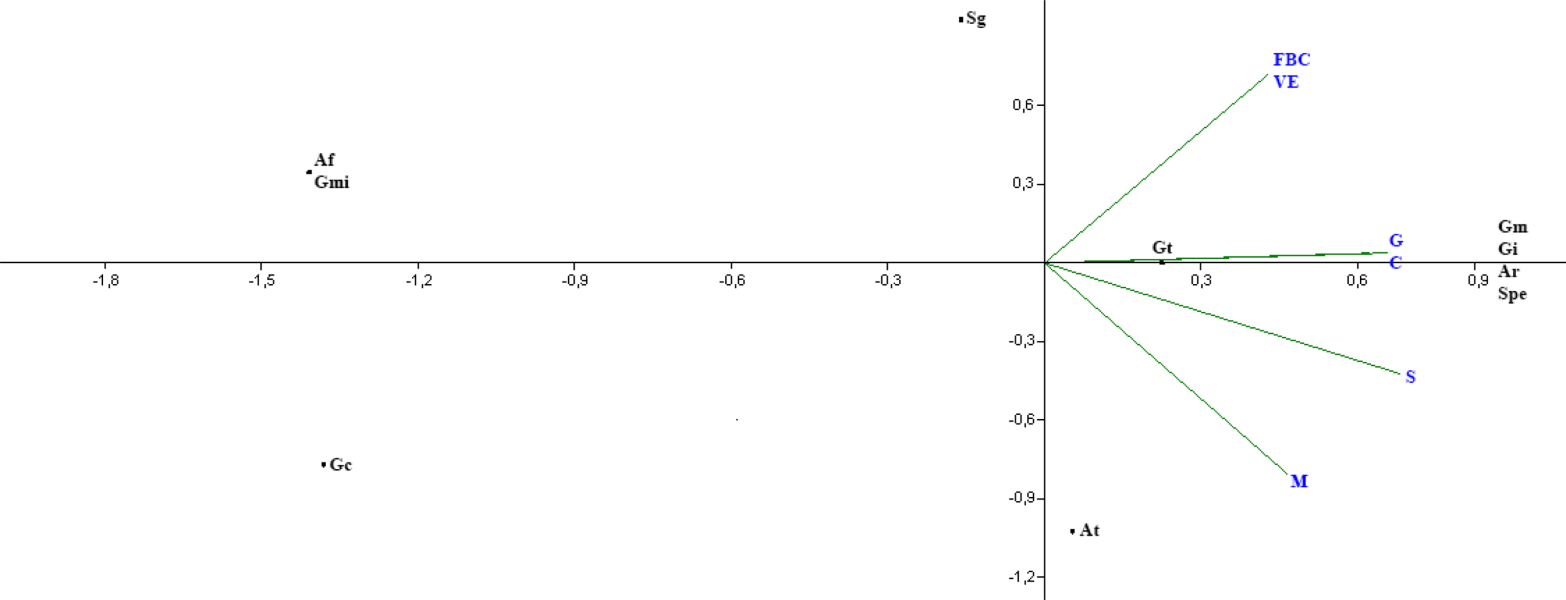
Analysis of principal components of the frequency of arbuscular mycorrhizal fungi species found in the rhizosphere of crotalaria (C), wild bean from Ceará (FBC), sorghum (S), Guandú (G), millet (M) and spontaneous vegetation (VE). (Af, *A. foveata*; At, *A. tuberculata*; Ar, *Ar. leptoticha*; Ge, *G. etunicatum*; Gm, *G. macrocarpum*; Gmi, *G. microaggregatum*; Gt, *G. tortuosum*; Gi, *Gigaspora* sp.; Sg, *S. gregaria*; Spe, *S. persica*).

When considering only the cover crops (**Figure 2**), the species *G. microaggregatum*, *S. gregaria*, *G. clavisporum*, and *A. foveata* present little proximity to the analyzed cover, which indicates the chance of the appearance of these species in the rhizosphere of the plants studied.

**Figure 3:**
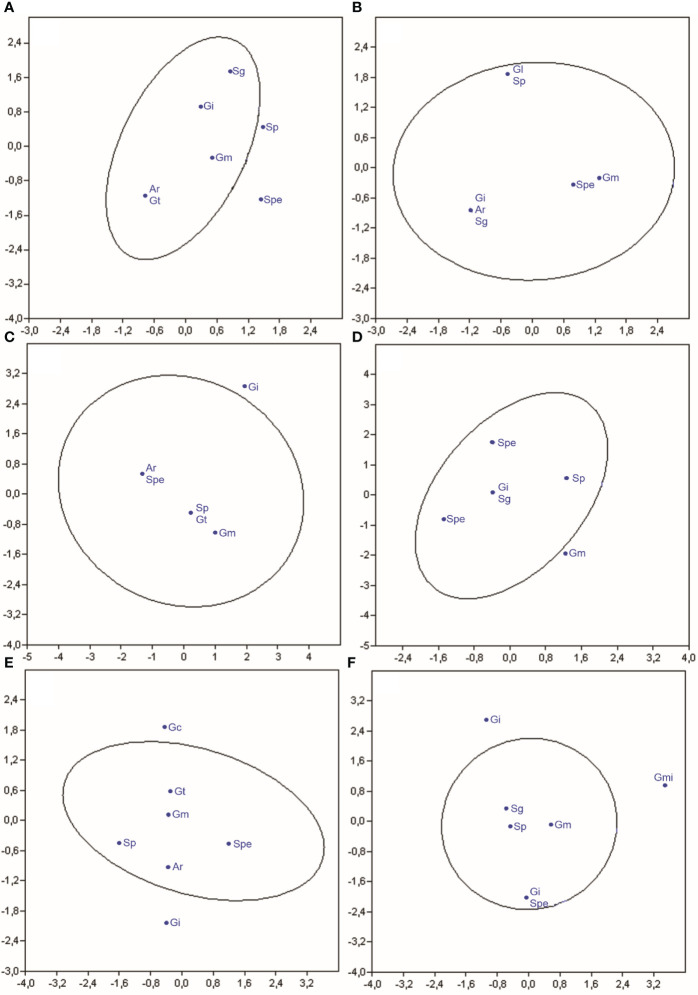
Canonical correspondence analysis of the frequency of arbuscular mycorrhizal fungal species in Crotalaria **(A)**, wild bean from Ceará **(B)**, sorghum **(C)**, Guandú **(D)**, millet **(E)**, and spontaneous vegetation **(F)** with and without nitrogen application. (Ar, *Ar. leptoticha*; Gc, *G. clarum*; Gm, *G. macrocarpum*; Gmi, *G. microaggregatum*; Gt, *G. tortuosum*; Gi, *Gigaspora sp*; Gl, *Glomus* sp.; Sg, *S. gregaria*; Sp, *S. pellucida*; Spe, *S. persica*).

The canonical correspondence analysis showed a correlation between the occurrence of the species of arbuscular mycorrhizal fungi identified with the cover crops with and without N application (**Figure 3**).

In the *C. juncea* rhizosphere, the frequency of AMF species did not differ in treatments with and without nitrogen application (**Figure 3A**). There was no differentiation of groups of organisms identified; therefore, the species present in treatments with nitrogen application were the same as those occurring in areas without nitrogen application. This behavior was also observed in the principal component analysis (**Figure 1**). *S. pellucida* and *S. persica* are not commonly found in the rhizosphere of this cover plant species, indicating causality in their appearance.

In the rhizosphere of wild beans from Ceará, the frequency of AMF species did not differ in treatments with and without nitrogen application (**Figure 3B**). There was no differentiation of groups of arbuscular mycorrhizal fungi identified between treatments with and without N application in the top dressing. Therefore, the species present in the plots with nitrogen are the same as those occurring in areas without nitrogen application. This behavior was also observed in the principal component analysis (**Figure 1**). All species of arbuscular mycorrhizal fungi identified are commonly found in the rhizosphere of wild beans.

The frequency of arbuscular mycorrhizal fungi found in the sorghum rhizosphere did not differ between treatments with and without nitrogen application in topdressing in the corn predecessor crop (**Figure 3C**). There was no differentiation of groups of arbuscular mycorrhizal fungi identified; therefore, the species present in the treatments with nitrogen were the same as those occurring in areas without nitrogen application coverage. This behavior was also observed in the principal component analysis (**Figure 1**). *Gigaspora* sp. was not found in the sorghum rhizosphere, indicating causality in its appearance. All other species of arbuscular mycorrhizal fungi identified in the rhizosphere of wild bean-do-ceará were commonly associated with this cover plant species.

The frequency of arbuscular mycorrhizal fungi found in the rhizosphere of Guandú did not differ between treatments with and without nitrogen topdressing (**Figure 3D**). There was no differentiation of groups of arbuscular mycorrhizal fungi identified; therefore, the species present in the plots with nitrogen were the same as those occurring in areas without nitrogen application. This behavior was also observed in the principal component analysis (**Figure 1**). *G. macrocarpum*. It is not commonly found in the rhizosphere of Guandú, indicating its appearance by chance. All other species of arbuscular mycorrhizal fungi found in the rhizosphere of Guandú are commonly associated with this cover plant species.

The frequency of arbuscular mycorrhizal fungi found in the millet rhizosphere did not differ between treatments with nitrogen application and treatments without application (**Figure 3E**). There was no differentiation of groups of arbuscular mycorrhizal fungi identified; therefore, the species present in treatments with nitrogen applied to the previous crop are the same as those occurring in areas without nitrogen application. This behavior was also observed in the principal component analysis (**Figure 1**). *G. macrocarpum* and *G. clavisporum* are not commonly found in the millet rhizosphere, indicating their occurrence by chance. All other arbuscular mycorrhizal fungi found in the millet rhizosphere are commonly associated with this cover plant species.

## Discussion

There are few studies that evaluate the influence of the effect of applying nitrogen fertilizer to corn crops in succession to cover crops on the mycorrhizal population. However, Benedetti et al. (2005) evaluated the spore density in soil under corn cultivation after sowing of millet, Crotalaria, Guandú, velvet bean, jack bean, and spontaneous vegetation (with the application of nitrogen in spontaneous vegetation) and without application of nitrogen in cover plants. The authors found much lower values in the soil under the corn crop in the treatments under spontaneous vegetation. The spore density in the corn crop varied between 39 and 50 spores 50 cm^−3,^and there was no significant difference between the cover crops. In the present work, the number of spores in the cover crops was evaluated, and they presented a very high number of spores ranging from 316 to 692 spores 50 cm^−3^, indicating that the use of these cover crops stimulates the formation of spores in the soil. The average number of spores (529.05 spores/50 g of soil) in the treatment with nitrogen application in topdressing in the corn crop was lower than that in the treatment without nitrogen application (567.83 spores/50 g of soil).

Ahiabor et al. (2007) evaluated spore density under the use of different cover crops and found spore density values in the soil under Crotalaria of 142 spores/50 g of soil in a savanna region in northern Ghana, lower than those obtained in the present work. When evaluating spore density in soybean and corn in succession to millet, crotalaria, and weeds, Angelini et al. (2012) found that the spore density was higher in corn after the cover crops were mentioned, with spore densities of 608, 414, and 306, respectively, when compared to soybeans from the same cover crops, with spore densities of 608, 414, and 306, respectively. 239, 287, and 259, respectively.

The verified spore density data can be considered high. This finding can be explained by the seasonal characteristics of the region where the plants were grown. The Cerrado is a region that has two well-defined climatic seasons, the dry season and the rainy season (Cardoso et al., 2015). The behavior of mycorrhizal fungi in Cerrado soils is closely linked to this seasonal variation (Vieira Junior et al., 2020). During the dry period, the mycorrhizal colonization rate is benefited from the need to support the water supply that fungi provide to plants, which in turn also promotes greater spore production by mycorrhizal fungi (Moura and Cabral, 2019).

Pedro et al. (2012) evaluated the influence of Guandú, *C. juncea*, millet, Guandú + millet, *C. juncea* + millet, and spontaneous vegetation with the application of four nitrogen rates (zero, 30, 60, and 90 kg ha^−1^) on the rate of mycorrhizal colonization and did not find significant differences between the treatments. The mycorrhizal colonization was between 58% and 63% in the soil under the different cover plants.

Despite the mycorrhizal colonization rate being similar among cover crops, other works, such as Angelini et al. (2012), found cover crops alter mycorrhizal colonization in the crop in succession. The authors verified that the mycorrhizal colonization rate in soybean and corn followed by millet, crotalaria, and weeds showed higher spore density in corn when followed by the mentioned cover crops, 98.7%, 95.5%, and 94.5%, respectively. Soybeans succeeded from the same cover crops, with 91.5%, 94.5%, and 80.7%, respectively.

Cordeiro et al. (2007) found spore density and mycorrhizal colonization in sorghum that were statistically lower than those found in areas with spontaneous vegetation and native cerrado. However, areas with grasses such as sorghum and millet showed higher values of colonization and spore density than areas with legumes such as Crotalaria, soybean, and jack bean. On the other hand, Silva et al. (2013) evaluated easily extractable glomalin in soil under *C. juncea*, jack bean, Guandú, velvet bean, and sorghum in succession to crops in agroforestry systems in the Cerrado and found that the highest levels of glomalin were observed in treatments with velvet bean and *C. juncea* in succession to maize and beans. Lower levels were observed in bean plots in succession to jack bean and Guandú.

Although it is not possible, with the analyses carried out in this work, to state that the quantified glomalin is totally produced by soil mycorrhizal fungi, its values constitute an important parameter of microbial activity in the soil (Purin and Rillig, 2007). Soil glomalin levels are altered by vegetation cover and soil management (Rillig and Steinberg, 2002; Borie et al., 2006; Roldán et al., 2007), as plants provide more photostimilation to AMF, favoring the production of glomalina by these microorganisms (Treseder and Turner, 2007). In addition, increases in soil carbon levels favor the formation and maintenance of aggregates (Winck et al., 2014). There are positive correlations between glomalin fractions and soil organic carbon content (Franzluebbers et al., 2000; Bird et al., 2002; Nichols and Wright, 2006). Several studies have also reported that glomalin production can be influenced by the type of land use, being lower in agricultural soils than in native soils (Rillig et al., 2003; Moura et al., 2019).

Soil turning destroys fungal hyphae and thus negatively influences AMF production of glomalin. Aggregate and glomalin t values, in areas even after 3 years of no-tillage system, were substantially lower when compared to values observed in areas of continuous pasture for 15 years (Wright and Upadhyaya, 1999). Species of the genera *Acaulospora*, *Archeospora*, Glomus, *Gigaspora*, and *Scutellospora* were identified in the rhizosphere of *C. juncea*, sweet bean, sorghum, Guandú, millet and spontaneous vegetation, with and without nitrogen application (**Table 5**). These same genera were found in other surveys carried out in Cerrado soil (Moura et al., 2019; Vieira Junior et al., 2020; dos Santos et al., 2021; Moura et al., 2022; dos Santos Lucas et al., 2022).

Benedetti et al. (2005) investigated the diversity of arbuscular mycorrhizal fungi in the rhizospheres of different cover crops and found that *A. delicata*, *A. spinosa*, *G. caledonium*, *G. etunicatum*, *G. tortuosum*, and *S. persica were* associated with the millet rhizosphere. *A. laevis*, *G. microaggregatum*, and *G. etunicatum were* associated with the rhizosphere of Guandú, *A. spinosa*, *A. scrobiculata*, *G. etunicatum*, *G. geosporum*, and *Glosmus* sp. in the *C. juncea* rhizosphere. The species *A. spinosa*, *G. microaggregatum*, *G. claraideum*, *G. etunicatum*, *G. geosporum*, and *Scutellospora* sp. were associated with spontaneous plants without nitrogen application. *A. spinosa*, *A. scrobiculata*, *G. microaggregatum*, *G. etunicatum*, *G. geosporum*, *S. gregaria*, and *S. persica* were detected in the rhizosphere of spontaneous plants with nitrogen application.

In this work, a result similar to that found by Benedetti et al. (2005) was obtained for the species *G. microaggregatum* that does not occur in the rhizosphere of cover plants, with or without the addition of N, but, only in the rhizosphere of spontaneous vegetation with the addition of N. Thus, it can be inferred that this species is adapted to soils with vegetation occurrence spontaneous in the area. However, further studies are needed on the dynamics of the diversity of this and other species of mycorrhizal fungi that occur in the rhizosphere of cover crops.

When analyzing the arbuscular mycorrhizal fungi community of cover crops in Cerrado agricultural systems, Angelini et al. (2012) observed the approximation of the frequent species in *C. juncea*, millet, and spontaneous plants, as well as in the data of this work presented in **Figure 1**. Li et al. (2007) analyzed the population of arbuscular mycorrhizal fungi in spontaneous vegetation of uncultivated areas and found a total of 47 species of AMF, of which 31 species belonged to the genus *Glomus*, eight to *Acaulospora*, six to *Scutellospora*, one to *Entrophospora*, and one to *Gigaspora.*

In soybean and corn rhizospheres cultivated in succession with *C. juncea*, Angelini et al. (2012) verified that the species *A. scrobiculata*, *A. mellea*, *Acaulospora* sp., *Gigaspora* sp., *G. tortuosum*, *S. scutata*, and *S. gregaria*. Angelini et al. (2012) found that the species *A. scrobiculata*, *A. scavata*, *A. mellea*, *Ar. leptoticha*, *Gigaspora* sp., *G. macrarpum*, *G. tortuosum*, *S. scutata*, and *S. gregaria*. The frequency of arbuscular mycorrhizal fungi found in the rhizosphere of spontaneous vegetation did not differ between treatments with and without nitrogen application in the top dressing (**Figure 3F**). There was no differentiation of groups of arbuscular mycorrhizal fungi identified; therefore, the species present in treatments with nitrogen in topdressing in corn are the same that occur in areas without application of nitrogen in topdressing. This behavior was also observed in the principal component analysis (**Figure 1**). *G. microaggregatum* and *G. tortuosum* are not commonly found in the soil rhizosphere under spontaneous vegetation, indicating their occurrence by chance. All other arbuscular mycorrhizal fungi found in the rhizosphere of wild plants are commonly associated with these cover plant species.

Massenssini et al. (2014) analyzed the correlation of 19 species of mycorrhizal fungi associated with the rhizosphere of spontaneous vegetation in soils of Minas Gerais and found no significant difference in root colonization between the studied areas. Angelini et al. (2012), in areas of spontaneous vegetation in the cerrado, verified the presence of *A. scrobiculata*, *A. scavata*, *A. mellea*, *Gigaspora* sp., *G. microaggregatum*, *G. tortuosum*, *S. scutata*, and *S. gregaria*.

## Conclusions

No influence of nitrogen application on soil spore density was verified, and among the cover crops studied, Guandu and Crotalaria presented the highest numbers of spores in the soil. There were no differences in the diversity of arbuscular mycorrhizal fungal species associated with the studied cover crops. The most frequent species associated with cover crops were *S. pellucida* and *S persica* in *C. juncea*; *Gigaspora* sp. in sorghum; *G. macrocarpum* in Guandu; *G. macrocarpum* and *G. clavisporum* in millet; and *G. microaggregatum* and *G. tortuosum* in spontaneous vegetation.

## Data availability statement

The original contributions presented in the study are included in the article/Supplementary Materials. Further inquiries can be directed to the corresponding author.

## Author contributions

JM—Project coordinator and responsible for writing. MR—Work advisor. MK—advisor on mycorrhizae. OJ—responsible for mycorrhizal analysis. WJ—responsible for the experimental area and experiments. AC—responsible for the experimental area and experiments. LS—responsible for laboratory analysis. RS—responsible for statistical analysis. SD and SS—text review. All authors contributed to the article and approved the submitted version.

## Conflict of interest

The authors declare that the research was conducted in the absence of any commercial or financial relationships that could be construed as a potential conflict of interest.

The handling editor declared a past collaboration with authors JM, RS, and SS.

## Publisher’s note

All claims expressed in this article are solely those of the authors and do not necessarily represent those of their affiliated organizations, or those of the publisher, the editors and the reviewers. Any product that may be evaluated in this article, or claim that may be made by its manufacturer, is not guaranteed or endorsed by the publisher.
